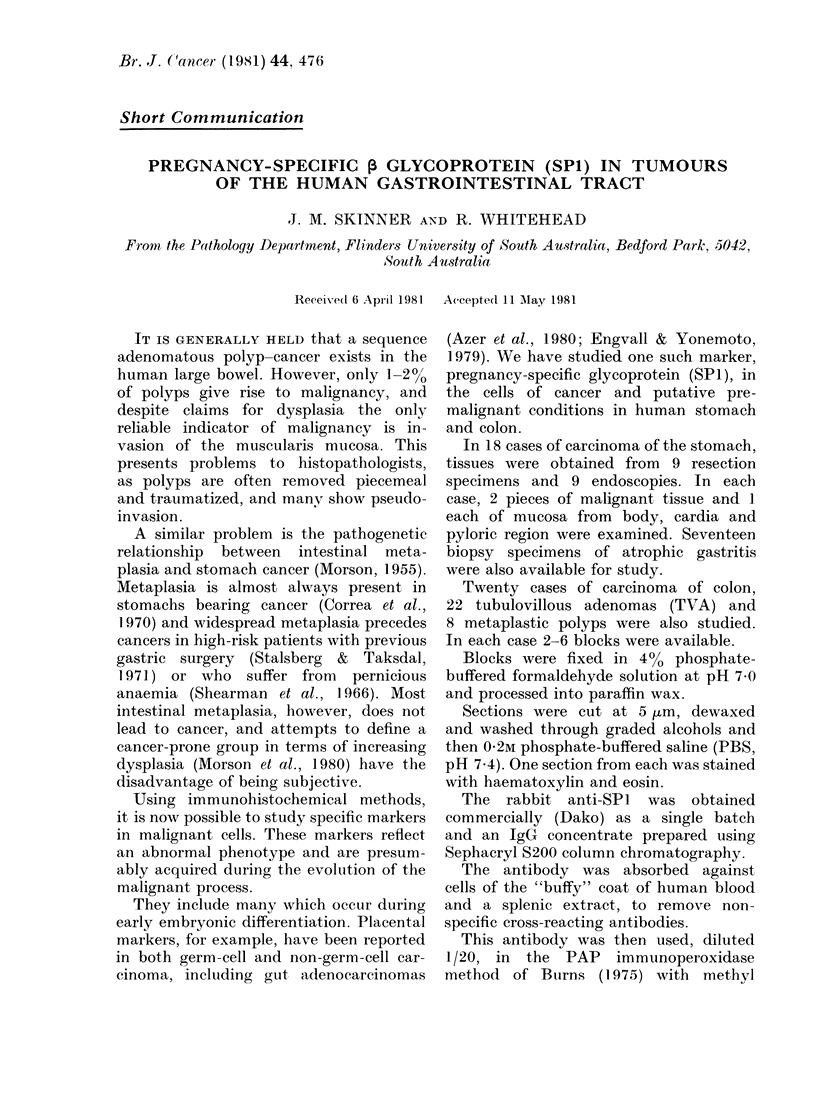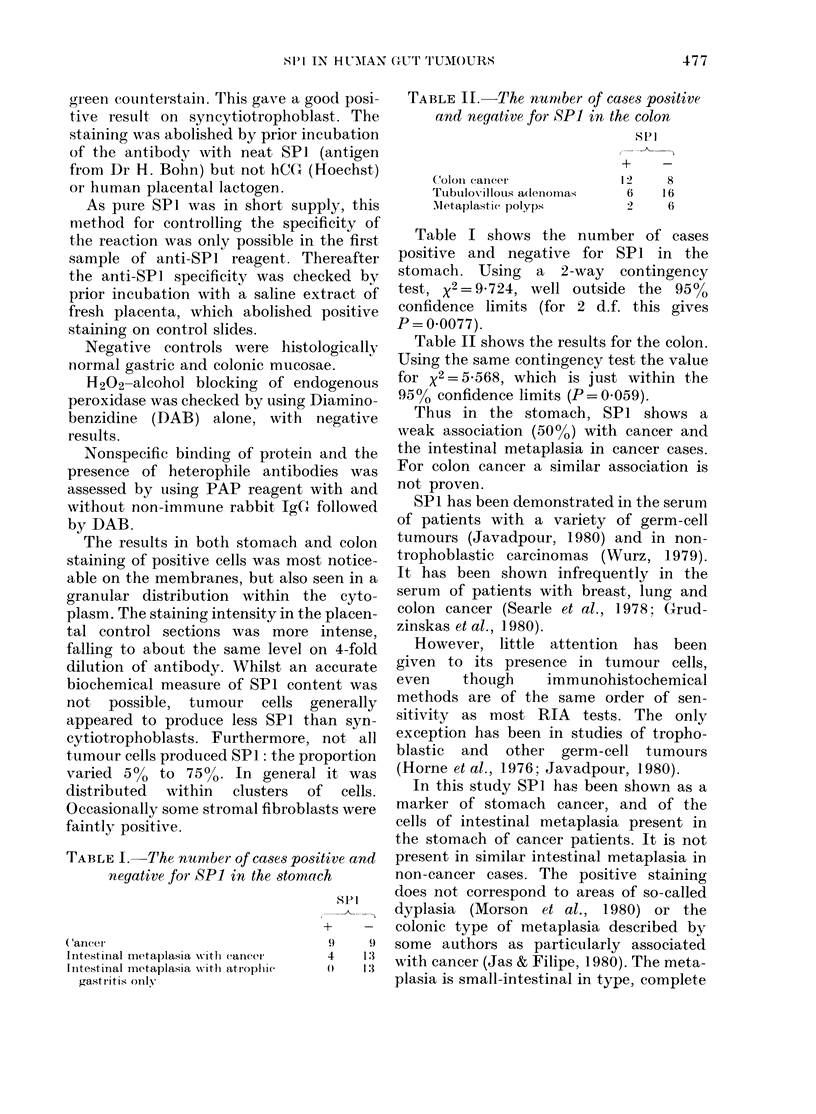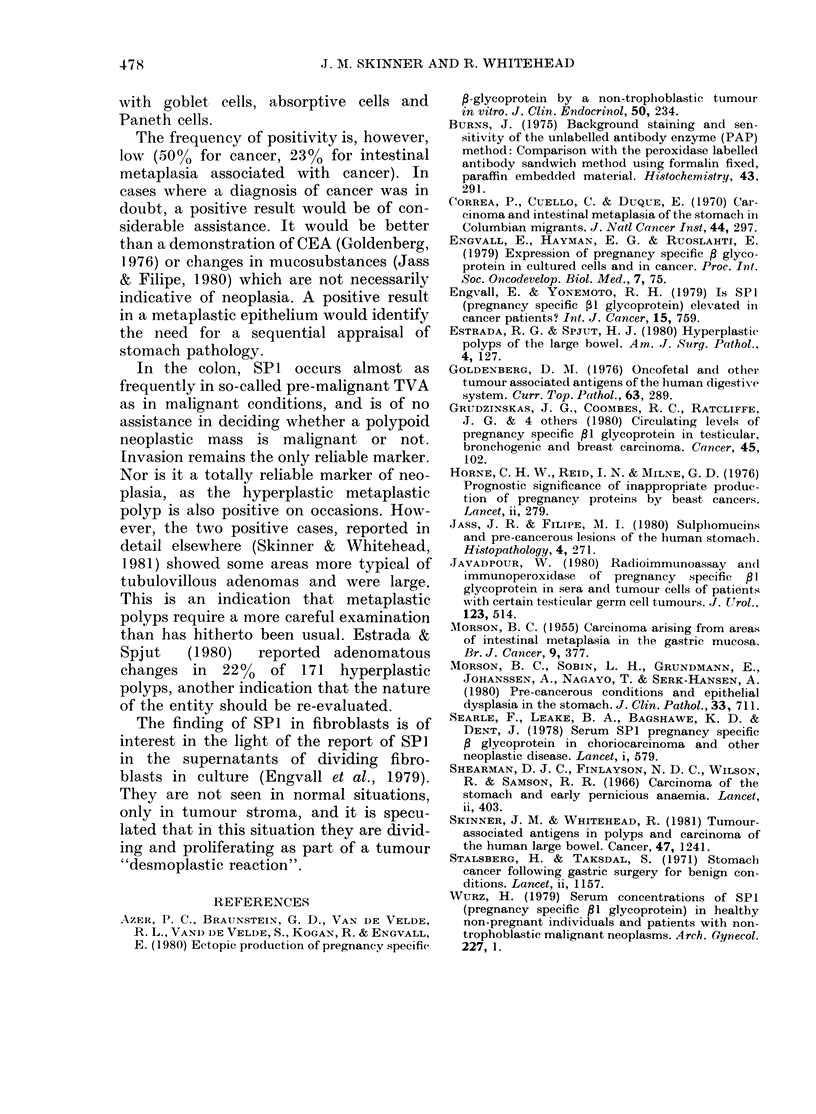# Pregnancy-specific beta glycoprotein (SP1) in tumours of the human gastrointestinal tract.

**DOI:** 10.1038/bjc.1981.210

**Published:** 1981-09

**Authors:** J. M. Skinner, R. Whitehead


*Br. J. Cancer* (1981) **44**, 476

***Short Communication***

### PREGNANCY-SPECIFIC β GLYCOPROTEIN (SP1) IN TUMOURS OF THE HUMAN GASTROINTESTINAL TRACT

J. M. SKINNER AND R. WHITEHEAD

*From the Pathology Department, Flinders University of South Australia, Bedford Park, 5042, South Australia*

Received 6 April 1981 Accepted 11 May 1981

IT IS GENERALLY HELD that a sequence adenomatous polyp–cancer exists in the human large bowel. However, only 1–2% of polyps give rise to malignancy, and despite claims for dysplasia the only reliable indicator of malignancy is invasion of the muscularis mucosa. This presents problems to histopathologists, as polyps are often removed piecemeal and traumatized, and many show pseudo-invasion.

A similar problem is the pathogenetic relationship between intestinal metaplasia and stomach cancer (Morson, 1955). Metaplasia is almost always present in stomachs bearing cancer (Correa *et al.*, 1970) and widespread metaplasia precedes cancers in high-risk patients with previous gastric surgery (Stalsberg & Taksdal, 1971) or who suffer from pernicious anaemia (Shearman *et al.*, 1966). Most intestinal metaplasia, however, does not lead to cancer, and attempts to define a cancer-prone group in terms of increasing dysplasia (Morson *et al.*, 1980) have the disadvantage of being subjective.

Using immunohistochemical methods, it is now possible to study specific markers in malignant cells. These markers reflect an abnormal phenotype and are presumably acquired during the evolution of the malignant process.

They include many which occur during early embryonic differentiation. Placental markers, for example, have been reported in both germ-cell and non-germ-cell carcinoma, including gut adenocarcinomas (Azer *et al.*, 1980; Engvall & Yonemoto, 1979). We have studied one such marker, pregnancy-specific glycoprotein (SP1), in the cells of cancer and putative pre-malignant conditions in human stomach and colon.

In 18 cases of carcinoma of the stomach, tissues were obtained from 9 resection specimens and 9 endoscopies. In each case, 2 pieces of malignant tissue and 1 each of mucosa from body, cardia and pyloric region were examined. Seventeen biopsy specimens of atrophic gastritis were also available for study.

Twenty cases of carcinoma of colon, 22 tubulovillous adenomas (TVA) and 8 metaplastic polyps were also studied. In each case 2–6 blocks were available.

Blocks were fixed in 4% phosphate-buffered formaldehyde solution at pH 7·0 and processed into paraffin wax.

Sections were cut at 5 μm, dewaxed and washed through graded alcohols and then 0·2M phosphate-buffered saline (PBS, pH 7·4). One section from each was stained with haematoxylin and eosin.

The rabbit anti-SP1 was obtained commercially (Dako) as a single batch and an IgG concentrate prepared using Sephacryl S200 column chromatography.

The antibody was absorbed against cells of the "buffy" coat of human blood and a splenic extract, to remove non-specific cross-reacting antibodies.

This antibody was then used, diluted 1/20, in the PAP immunoperoxidase method of Burns (1975) with methyl green counterstain. This gave a good positive result on syncytiotrophoblast. The staining was abolished by prior incubation of the antibody with neat SP1 (antigen from Dr H. Bohn) but not hCG (Hoechst) or human placental lactogen.

As pure SP1 was in short supply, this method for controlling the specificity of the reaction was only possible in the first sample of anti-SP1 reagent. Thereafter the anti-SP1 specificity was checked by prior incubation with a saline extract of fresh placenta, which abolished positive staining on control slides.

Negative controls were histologically normal gastric and colonic mucosae.

$H_2O_2$–alcohol blocking of endogenous peroxidase was checked by using Diaminobenzidine (DAB) alone, with negative results.

Nonspecific binding of protein and the presence of heterophile antibodies was assessed by using PAP reagent with and without non-immune rabbit IgG followed by DAB.

The results in both stomach and colon staining of positive cells was most noticeable on the membranes, but also seen in a granular distribution within the cytoplasm. The staining intensity in the placental control sections was more intense, falling to about the same level on 4-fold dilution of antibody. Whilst an accurate biochemical measure of SP1 content was not possible, tumour cells generally appeared to produce less SP1 than syncytiotrophoblasts. Furthermore, not all tumour cells produced SP1: the proportion varied 5% to 75%. In general it was distributed within clusters of cells. Occasionally some stromal fibroblasts were faintly positive.

TABLE I.—*The number of cases positive and negative for SP1 in the stomach*

| | SP1 | |
|---|---|---|
| | + | − |
| Cancer | 9 | 9 |
| Intestinal metaplasia with cancer | 4 | 13 |
| Intestinal metaplasia with atrophic gastritis only | 0 | 13 |

TABLE II.—*The number of cases positive and negative for SP1 in the colon*

| | SP1 | |
|---|---|---|
| | + | − |
| Colon cancer | 12 | 8 |
| Tubulovillous adenomas | 6 | 16 |
| Metaplastic polyps | 2 | 6 |

Table I shows the number of cases positive and negative for SP1 in the stomach. Using a 2-way contingency test, $\chi^2 = 9{\cdot}724$, well outside the 95% confidence limits (for 2 d.f. this gives $P = 0{\cdot}0077$).

Table II shows the results for the colon. Using the same contingency test the value for $\chi^2 = 5{\cdot}568$, which is just within the 95% confidence limits ($P = 0{\cdot}059$).

Thus in the stomach, SP1 shows a weak association (50%) with cancer and the intestinal metaplasia in cancer cases. For colon cancer a similar association is not proven.

SP1 has been demonstrated in the serum of patients with a variety of germ-cell tumours (Javadpour, 1980) and in non-trophoblastic carcinomas (Wurz, 1979). It has been shown infrequently in the serum of patients with breast, lung and colon cancer (Searle *et al.*, 1978; Grudzinskas *et al.*, 1980).

However, little attention has been given to its presence in tumour cells, even though immunohistochemical methods are of the same order of sensitivity as most RIA tests. The only exception has been in studies of trophoblastic and other germ-cell tumours (Horne *et al.*, 1976; Javadpour, 1980).

In this study SP1 has been shown as a marker of stomach cancer, and of the cells of intestinal metaplasia present in the stomach of cancer patients. It is not present in similar intestinal metaplasia in non-cancer cases. The positive staining does not correspond to areas of so-called dyplasia (Morson *et al.*, 1980) or the colonic type of metaplasia described by some authors as particularly associated with cancer (Jas & Filipe, 1980). The metaplasia is small-intestinal in type, complete with goblet cells, absorptive cells and Paneth cells.

The frequency of positivity is, however, low (50% for cancer, 23% for intestinal metaplasia associated with cancer). In cases where a diagnosis of cancer was in doubt, a positive result would be of considerable assistance. It would be better than a demonstration of CEA (Goldenberg, 1976) or changes in mucosubstances (Jass & Filipe, 1980) which are not necessarily indicative of neoplasia. A positive result in a metaplastic epithelium would identify the need for a sequential appraisal of stomach pathology.

In the colon, SP1 occurs almost as frequently in so-called pre-malignant TVA as in malignant conditions, and is of no assistance in deciding whether a polypoid neoplastic mass is malignant or not. Invasion remains the only reliable marker. Nor is it a totally reliable marker of neoplasia, as the hyperplastic metaplastic polyp is also positive on occasions. However, the two positive cases, reported in detail elsewhere (Skinner & Whitehead, 1981) showed some areas more typical of tubulovillous adenomas and were large. This is an indication that metaplastic polyps require a more careful examination than has hitherto been usual. Estrada & Spjut (1980) reported adenomatous changes in 22% of 171 hyperplastic polyps, another indication that the nature of the entity should be re-evaluated.

The finding of SP1 in fibroblasts is of interest in the light of the report of SP1 in the supernatants of dividing fibroblasts in culture (Engvall *et al.*, 1979). They are not seen in normal situations, only in tumour stroma, and it is speculated that in this situation they are dividing and proliferating as part of a tumour "desmoplastic reaction".

### REFERENCES

AZER, P. C., BRAUNSTEIN, G. D., VAN DE VELDE, R. L., VAND DE VELDE, S., KOGAN, R. & ENGVALL, E. (1980) Ectopic production of pregnancy specific β-glycoprotein by a non-trophoblastic tumour *in vitro*. *J. Clin. Endocrinol*, **50**, 234.

BURNS, J. (1975) Background staining and sensitivity of the unlabelled antibody enzyme (PAP) method: Comparison with the peroxidase labelled antibody sandwich method using formalin fixed, paraffin embedded material. *Histochemistry*, **43**, 291.

CORREA, P., CUELLO, C. & DUQUE, E. (1970) Carcinoma and intestinal metaplasia of the stomach in Columbian migrants. *J. Natl Cancer Inst*, **44**, 297.

ENGVALL, E., HAYMAN, E. G. & RUOSLAHTI, E. (1979) Expression of pregnancy specific β glycoprotein in cultured cells and in cancer. *Proc. Int. Soc. Oncodevelop. Biol. Med.*, **7**, 75.

Engvall, E. & YONEMOTO, R. H. (1979) Is SP1 (pregnancy specific β1 glycoprotein) elevated in cancer patients? *Int. J. Cancer*, **15**, 759.

ESTRADA, R. G. & SPJUT, H. J. (1980) Hyperplastic polyps of the large bowel. *Am. J. Surg. Pathol.*, **4**, 127.

GOLDENBERG, D. M. (1976) Oncofetal and other tumour associated antigens of the human digestive system. *Curr. Top. Pathol.*, **63**, 289.

GRUDZINSKAS, J. G., COOMBES, R. C., RATCLIFFE, J. G. & 4 others (1980) Circulating levels of pregnancy specific β1 glycoprotein in testicular, bronchogenic and breast carcinoma. *Cancer*, **45**, 102.

HORNE, C. H. W., REID, I. N. & MILNE, G. D. (1976) Prognostic significance of inappropriate production of pregnancy proteins by beast cancers. *Lancet*, ii, 279.

JASS, J. R. & FILIPE, M. I. (1980) Sulphomucins and pre-cancerous lesions of the human stomach. *Histopathology*, **4**, 271.

JAVADPOUR, W. (1980) Radioimmunoassay and immunoperoxidase of pregnancy specific β1 glycoprotein in sera and tumour cells of patients with certain testicular germ cell tumours. *J. Urol.*, **123**, 514.

MORSON, B. C. (1955) Carcinoma arising from areas of intestinal metaplasia in the gastric mucosa. *Br. J. Cancer*, **9**, 377.

MORSON, B. C., SOBIN, L. H., GRUNDMANN, E., JOHANSSEN, A., NAGAYO, T. & SERK-HANSEN, A. (1980) Pre-cancerous conditions and epithelial dysplasia in the stomach. *J. Clin. Pathol.*, **33**, 711.

SEARLE, F., LEAKE, B. A., BAGSHAWE, K. D. & DENT, J. (1978) Serum SP1 pregnancy specific β glycoprotein in choriocarcinoma and other neoplastic disease. *Lancet*, i, 579.

SHEARMAN, D. J. C., FINLAYSON, N. D. C., WILSON, R. & SAMSON, R. R. (1966) Carcinoma of the stomach and early pernicious anaemia. *Lancet*, ii, 403.

SKINNER, J. M. & WHITEHEAD, R. (1981) Tumour-associated antigens in polyps and carcinoma of the human large bowel. Cancer, **47**, 1241.

STALSBERG, H. & TAKSDAL, S. (1971) Stomach cancer following gastric surgery for benign conditions. *Lancet*, ii, 1157.

WURZ, H. (1979) Serum concentrations of SP1 (pregnancy specific β1 glycoprotein) in healthy non-pregnant individuals and patients with non-trophoblastic malignant neoplasms. *Arch. Gynecol.* **227**, 1.